# Development of a New Microextraction Fiber Combined to On-Line Sample Stacking Capillary Electrophoresis UV Detection for Acidic Drugs Determination in Real Water Samples

**DOI:** 10.3390/ijerph14070739

**Published:** 2017-07-07

**Authors:** Maria Espina-Benitez, Lilia Araujo, Avismelsi Prieto, Alberto Navalón, José Luis Vílchez, Paola Valera, Ana Zambrano, Vincent Dugas

**Affiliations:** 1 Laboratory of Analytical Chemistry and Electrochemistry, Faculty of Engineering, University of Zulia, P.O. Box 4011-A-526, Maracaibo 4005, Venezuela; laraujo@fing.luz.edu.ve (L.A.); aprieto@fing.luz.edu.ve (A.P.); rosselyv@gmail.com (P.V.); luzdaly_zam@hotmail.com (A.Z.); 2 Université de Lyon, CNRS, Université Claude Bernard Lyon 1, ENS de Lyon, Institut des Sciences Analytiques, UMR 5280, 5 rue de la Doua, F-69100 VILLEURBANNE, France; vincent.dugas@univ-lyon1.fr; 3 Research Group of Analytical Chemistry and Life Sciences, Department of Analytical Chemistry, Faculty of Sciences, University of Granada, Campus of Fuentenueva, E-18071 Granada, Spain; anavalon@ugr.es (A.N.); jvilchez@ugr.es (J.L.V.)

**Keywords:** acidic drugs, capillary zone electrophoresis, solid-phase microextraction, porapak Q

## Abstract

A new analytical method coupling a (off-line) solid-phase microextraction with an on-line capillary electrophoresis (CE) sample enrichment technique was developed for the analysis of ketoprofen, naproxen and clofibric acid from water samples, which are known as contaminants of emerging concern in aquatic environments. New solid-phase microextraction fibers based on physical coupling of chromatographic supports onto epoxy glue coated needle were studied for the off-line preconcentration of these micropollutants. Identification and quantification of such acidic drugs were done by capillary zone electrophoresis (CZE) using ultraviolet diode array detection (DAD). Further enhancement of concentration sensitivity detection was achieved by on-line CE “acetonitrile stacking” preconcentration technique. Among the eight chromatographic supports investigated, Porapak Q sorbent showed higher extraction and preconcentration capacities. The screening of parameters that influence the microextraction process was carried out using a two-level fractional factorial. Optimization of the most relevant parameters was then done through a surface response three-factor Box-Behnken design. The limits of detection and limits of quantification for the three drugs ranged between 0.96 and 1.27 µg∙L^−1^ and 2.91 and 3.86 µg∙L^−1^, respectively. Recovery yields of approximately 95 to 104% were measured. The developed method is simple, precise, accurate, and allows quantification of residues of these micropollutants in Genil River water samples using inexpensive fibers.

## 1. Introduction

**Figure 1:**
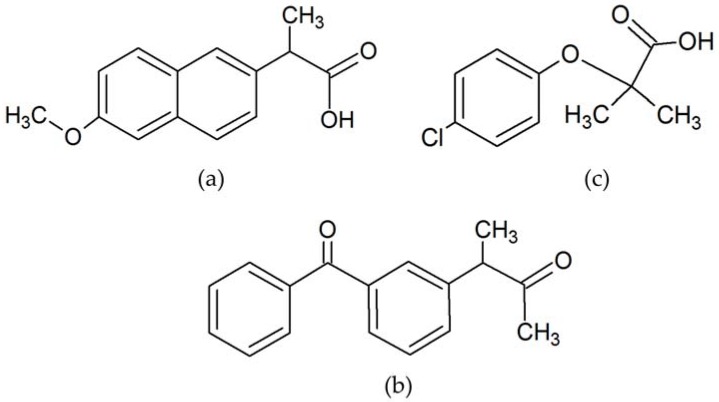
Molecular structure of: (**a**) naproxen; (**b**) ketoprofen; and (**c**) clofibric acid.

In recent years, a very large number of micropollutants conformed by pharmaceutical and product care products (PPCPs), known as contaminants of emerging concern (CECs), have been detected in aquatic environments. Among these CECs, acidic drugs such as ketoprofen, naproxen and clofibric acid are among the most used pharmaceutical products in human and veterinary medicine (Figure 1). These substances of high polarity are highly soluble in water, thus, after excretion from humans and animals and incorporation into the wastewater, they are not fully eliminated by treatment plants and the probability of reaching natural water bodies and entering the food chain through fish and other aquatic fauna and flora is increased [1,2,3]. The disposal of PPCPs wastes raise the need to monitor their concentration in the environment, especially in natural water bodies. This has become a topic of interest in different research worldwide, since its consequences over aquatic life and quality of life of human beings are unknown [3,4,5].

In order to control and monitor the concentration of these new pollutants, there is a need to develop accurate and precise analytical methods, sufficiently sensitive and with short-time analysis to identify and quantify drugs in aqueous samples [6]. Gas chromatography (GC) [7,8,9] and high performance liquid chromatography (HPLC) have been commonly used for the determination of acidic drugs [6,10,11,12,13,14]. In recent years, Capillary Electrophoresis (CE) has also been used for the separation of acidic drugs. This powerful separation technique is more ecological and economical than chromatography-based methods. Indeed, it is a miniaturized technique using low volume of aqueous background electrolytes. However, the small optical path lengths related to the in-capillary detection (50–75 µm) reduces the sensitivity of the technique. Quantitative analysis at the trace concentration level requires coupling CE to preconcentration techniques (electrophoresis-based or chromatography-based techniques) to increase the sensitivity [15,16,17,18,19] when diode array detection (DAD) is used. Among the alternatives, there are on-line and off-line preconcentration techniques such as acetonitrile (ACN) stacking CE and solid-phase microextraction (SPME). 

SPME has the advantages of being a quick and simple technique that requires no solvents, and exhibits good linearity and sensitivity, when compared with conventional techniques such as solid-phase extraction (SPE). However, it has certain requirements that are not so attractive to researchers, such as high cost, fragility and limited lifetime of commercial fibers due to the experimental conditions, as they tend to degrade as they are used. These drawbacks have led to the development of various technologies and techniques in order to prepare laboratory-made fibers, but most of the procedures are complicated and time consuming [9,11,19,20,21,22,23,24,25,26,27,28,29,30].

Regarding the ACN stacking, it has been found that the use of this on-line preconcentration strategy provides several advantages such as: lower conductivity and therefore improved current stability, focusing of solutes as the organic solvent and salt ions are present to induce an “isotachophoresis mechanism”, and enhancement of sensitivity and concentration factor compensation due to the large sample volume injection [16,17,31,32]. To our knowledge, this approach has been used for the analysis of ketoprofen with CE [33], however the simultaneous analysis of acidic drugs using ACN stacking has not yet been investigated.

Furthermore, SPME was used as an off-line preconcentration technique for the analysis of acidic drugs coupled to HPLC and GC in order to lower their limits of detection and quantification in complex environmental samples [7,9,20,21,22,23]. Herein, we propose to develop a non-commercial SPME fiber for the off-line purification and preconcentration of acidic drugs in water samples coupled to an on-line ACN stacking CE-DAD analysis. This original coupling strategy leads to enhance sensitivity in CE analysis using conventional UV detection. In addition to its environmentally-friendly feature, this strategy is an affordable alternative approach for the analysis of acidic drugs in natural water bodies.

This article describes the development, optimization and validation of an original solid-phase microextraction “fiber-like” support allowing the off-line preconcentration of highly soluble acidic drugs coupled to on-line capillary electrophoresis sample enrichment (acetonitrile stacking preconcentration). Baseline separation of drugs was successfully obtained using 31.25 mM phosphate buffer at pH 8.75 within 12 min. The microextraction fibers were easily prepared by a direct physical deposition of chromatographic supports onto epoxy glue coated needle. This procedure was used to prepare different microextraction supports. Optimization of the experimental parameters of the solid-phase microextraction step was performed following a methodological approach based on design of experiments. The overall method was then applied to the simultaneous determination of ketoprofen, naproxen and clofibric acid from Genil River water sample.

## 2. Materials and Methods

### 2.1. Reagents

All reagents were of analytical grade and all solvents were HPLC grade. Chromatographic supports, ketoprofen, naproxen and clofibric acid were purchased from Sigma-Aldrich (Sigma-Aldrich, Madrid, Spain). From these, the standard solutions of acidic drugs were prepared. Enrofloxacin was used as internal standard at a concentration of 1 mg∙L^−1^, prepared from a stock solution of 1000 mg∙L^−1^ dissolved in acetonitrile. All aqueous solutions were prepared using >18 MΩ-cm water. Polymimide-coated fused-silica capillaries (75 µm inner diameter) were purchased from Polymicro Technologies (Polymicro Technologies, Phoenix, AZ, USA). 

Stock solutions of 1000 mg∙L^−1^ of ketoprofen, naproxen and clofibric acid were prepared by dissolving the pure reagents in acetonitrile. Standard solutions were prepared by the dilution of the stocks with ultrapure water at pH 2, previously acidified with HCl solution, and vacuum filtered with a 0.45 μm cellulose ester filter membrane (Advantec MFS Inc., Dublin, CA, USA).

### 2.2. Preparation of the Microextraction Fibers

**Figure 2:**
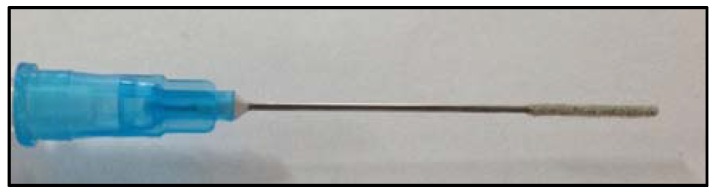
Laboratory-made SPME fiber. SPME solid–phase microextraction.

For preparing the fiber, the needle of a commercial disposable syringe (23G Gaesca; 2.54 cm × 0.6 mm) was used as a support. The needle tip was cut and the surface was sanded. Then, 1 cm of the tip was coated with epoxy resin (Araldite 7070) and immersed in a vial containing Porapak Q sorbent, then allowed to dry for 24 h in a desiccator. Subsequently, the fiber was washed with methanol 99.9% purity for 25 min and with deionized water for 15 min in a vial with continuous stirring. A typical laboratory-made solid-phase microextraction (SPME) fiber with Porapak Q as sorbent is shown in Figure 2.

### 2.3. Apparatus and Separation Conditions

The determination of the drugs was performed using a Capillary Electrophoresis Agilent model G1600AX equipped with diode array detector (DAD). The storage and processing of capillary electrophoresis (CE) with DAD detection (CE-DAD) system data was performed using the Chemstation software. The separations were carried out with a fused silica capillary of 62 cm total length (54 cm effective length), 75 µm inner diameter and 375 µm outer diameter. Analyses were performed at 20 kV and 25 °C. Naproxen detection was performed at 228 nm, while the ketoprofen and clofibric acid detection was performed at 203 nm. Samples were introduced into the anodic end of the capillary by hydrodynamic injection at 50 mbar for 50 s. 

The background electrophoretic solution used for the separation was composed of 31.25 mM sodium phosphate (NaH_2_PO_4_), 8% acetonitrile at pH 8.75. This solution was prepared by mixing 25 mL of 125 mM sodium dihydrogen phosphate solution and 8 mL of acetonitrile, adjusting the pH of the solution to 8.75 with NaOH solution and diluting this solution with ultrapure water in a 100 mL volumetric flask and filtered with the aid of vacuum filtration equipment (Cole Parmer) and a 0.45 μm cellulose ester filter membrane. The on-line preconcentration by acetonitrile stacking was carried out by dissolving samples and standards in electrophoretic solution/acetonitrile 25/75 (*v/v*) solution.

New fused silica capillaries were activated by flushing a 1 M NaOH solution for 10 min, then a 0.2 M NaOH solution for 10 min followed by a flush of deionized water for 5 min, at 2-bar pressure. At the beginning of each working day, the capillary was rinsed with a 0.2 M NaOH solution, then with deionized water and finally electrophoretic solution, for 5 min each. Between runs, preconditioning was performed at 3 bar with 1 M NaOH for 1 min, deionized water for 1 min and electrophoretic solution for 2 min, and a post-conditioning with deionized water for 2 min at 3 bar.

An internal standard (enrofloxacin) was added to the eluted solution before CE experiment. This is helpful to compensate the experimental errors that may influence the precision of the analytical response, such as the injection volume and the electroosmotic flow between each run, by normalization of the peak area of each analyte.

### 2.4. Solid-Phase Microextraction Protocol

For the solid-phase microextraction, an 8.0 mL vial was used, in which 5.0 mL of samples or standard solutions with controlled pH at 2.0 were placed. Subsequently, a magnetic stirrer and Porapak Q fiber were placed in the vial, leaving it in direct contact with the solution, with a stirring speed of 600 rpm. After 45 min of extraction, the fiber was dried under nitrogen stream and then the elution/desorption of analytes were performed by immersion of the fiber in 100 µL of 22.5 mM phosphate buffer (pH 8.75)/acetonitrile 25/75 (*v/v*) solution assisted by sonication for 4 min. This desorption solution was then directly injected in the capillary electrophoresis for further separation and quantification of acidic drugs. The fiber was washed with water and MeOH during 10 min each and dried under nitrogen stream between analyses.

### 2.5. Design of Experiment

In order to obtain optimized conditions for extraction, a fractional factorial experimental design was used to evaluate the preliminary significance of the variables, as well as the interactions between them. The variables investigated were: sample volume, salt concentration; sorbent type, stirring speed, extraction temperature, extraction time and desorption time. All variables were evaluated at two levels, low (denoted as −1) and high (denoted as +1). The data were processed using the Statgraphics Centurion XVI computer program.

The levels for the experimental design are summarized in Table 1. The concentration of the analytes was kept constant during the experiments at 37.5, 50.0 and 75.0 µg∙L^−1^ for ketoprofen, naproxen and clofibric acid, respectively.

The significant variables indicated by the Pareto chart were optimized using a Box-Behnken design, which is a response surface methodology, based on a three-level factorial design. The variables and levels used for the Box-Behnken design are shown in Table 2.

### 2.6. Water Samples

Water samples from the Genil river of Granada, Spain were collected upstream of the drinking water treatment plant in 5 L-glass bottles previously washed with sulphochromic mixture and dried in the oven at 120 °C for 48 h. Then, 2.5 L portions were taken from two different zones of the river and were added to the same container to form a composite sample. Upon completion of collection, 1 L of the sample was pH-adjusted at 2 with 6 M and 1 M HCl, filtered through a 0.45 μm porous membrane filter and then stored at 4 °C until analysis. 

## 3. Results

### 3.1. Optimization of Electrophoretic Separation

According to the literature, the electrophoretic solutions most used for the analysis of nonsteroidal anti-inflammatory drugs have been borate, acetate and phosphate, in a pH range of 7 to 9, since acidic drugs are found as anions thanks to their pKa values between 3 and 7 under these conditions [15,18]. Therefore, the electrophoretic separation of ketoprofen, naproxen and clofibric acid (with pKa of 4.0, 4.2 and 4.4, respectively) was performed using phosphate buffer as background electrolyte. The separation was investigated in a pH range spanning from 7.8 to 9.2 and in a phosphate buffer concentration range between 25.0 and 62.5 mM (data not shown). All acidic drugs were baseline separated using phosphate buffer at pH 8.75. At this pH, the analytes are negatively charged and migrate in the opposite direction of electroosmotic flow. The optimum buffer concentration was 31.25 mM, which provided a good compromise between peak shape, resolution and intensity of the electric current (heat production). Addition of 8% (*v/v*) of acetonitrile as organic modifier to the phosphate buffer improved the separation in terms of resolution. Optimal electrophoretic separation was performed at +20 kV. Higher voltages did not improve resolution due to excessive heat production and thus band-broadening effect.

### 3.2. Optimization of the On-Line Preconcentration by “Acetonitrile Stacking”

In order to increase the sensitivity of detection of acidic drugs, an on-line capillary electrophoresis (CE) sample preconcentration technique was investigated. This was obtained by injecting hydrodynamically a controlled (and large) volume of a low conductivity sample inside of a capillary filled of high conductivity background electrolyte (BGE). Through voltage application, electric field generated through the different zones (sample zone and BGE zone) is governed by the conductivity of the media. In a uniform inner diameter capillary, electric field is inversely (directly) proportional to the conductivity (resistance) of the zone. Knowing that electrophoretic migration is directly proportional to the electric field, the difference of conductivity between the sample and BGE zones determine the difference of velocity of analytes between the two zones. As a result analytes in the low conductivity sample zone migrate faster than in the BGE zone. Thus, upon voltage application, analytes migrate rapidly towards the BGE boundary where they stack due to lower migration velocity. In acetonitrile stacking CE, acetonitrile is added to sample to lower the conductivity of the sample zone and enhance the stacking effect and thus the preconcentration factor [34].

In this case, both the conductivity of the sample zone as the injection volume needed to be carefully optimized. With the aim of obtaining a sample solution of low conductivity acetonitrile is added to phosphate buffered sample solutions in a 25/75 (*v/v*) sample/acetonitrile ratio. This sample/acetonitrile ratio was selected as it leads the better sensitivity and robustness (current stability) compromise. Optimization of the injection volume was performed by increasing the injection time from 10 and 100 s at 50 mbar pressure. Since ketoprofen is primarily detected at 203 nm and naproxen at 228 nm, optimal injection time was determined according to the resolution measurement of this critical pair. A time of injection of 50 s led to a resolution of 1.21. Higher injection time led to partial overlapping of naproxen and clofibric acid peaks as shown in Table 3. The volume of injection using such injection parameters (50 s of injection time at 50 mbar) represents approximately 11% of the capillary volume. It is worth noting that injection time does not affect significantly migration times of analytes, while generating a 6–12-fold increase in surface area of analyte peaks. Finally, the optimal chosen conditions for on-line preconcentration are: (i) addition of acetonitrile to aqueous samples in a 25:75 (*v/v*) ratio, respectively; and (ii) injection time set at 50 mbar for 50 s.

### 3.3. Optimization of Solid-Phase Microextractionstep through a Design of Experiment Approach

In a preliminary study, coated fibers with graphitized carbon, Chromosorb P, LiChrolut, C_18_-functionalized silica particles and Porapak N, R, Q and QS sorbents, were prepared and compared with respect to extraction efficiency of acidic drugs, as discussed in Supplementary Materials (Figure S1). Fibers were exposed to standard solutions of acidic drugs at pH 2 (to ensure their unprotonated form) for 45 min. Elution (desorption) of extracted analytes from solid-phase microextraction (SPME) fibers was performed using a 100 µL of 22.5 mM phosphate buffer (pH 8.75)/acetonitrile 25/75 (*v/v*) solution assisted by sonication. This mixture is a good compromise of suitable polarity (and pH) for efficient desorption of analytes from fibers and low conductivity for on-line CE preconcentration. 

Regarding the evaluation of different types of sorbent, C_18_-functionalized silica particles were chosen as the reference sorbent. Two different thicknesses of coating layer for such C_18_ silica particles were tested: a “thin” one, i.e., 0.9 millimeters and a “thick” one, i.e., 1.5 millimeters. Moreover, we decided to study other types of sorbent in terms of cost, efficiency and specificity as: graphitized carbon, LiChrolut, Chromosorb P and a serie of Porapak materials (Q, QS, R and N). 

The graphitized carbon fibers loss part of their coating during the desorption step. This behavior is attributed to the large particle size, which confer it a low mechanical resistance. Capillary clogging during the capillary zone electrophoresis (CZE) experiments was observed using the thick C_18_ coating fiber. This was also attributed to released particles from the coating when shocks occur between the fiber and the vial insert during sonication. Therefore, both fibers were discarded from the group of fiber sorbents further investigated.

Regarding the remaining sorbents, no extraction was observed with the LiChrolut (polymer sorbent with high adsorption capacity for polar compounds) and Chromosorb P (silica-based particles) sorbents whereas the C_18_ silica particles and Porapak fibers (porous polymer-based sorbents sold for separation of apolar compounds) allowed extraction as presented Figure S1. It seems that interactions of these materials for the selected analytes are related to the “apolar” character of the coating. Indeed, extraction was realized at pH 2, solutes are under their “hydrophobic” molecular form.

Moreover, Figure S1 shows that between the Porapak serie, non-specific interactions are more pronounced with the types N, R and QS. In conclusion, C_18_ functionalized silica and Porapak Q particles were selected for the SPME optimization process

A first set of experiments was implemented in order to screen experimental variables that influence the solid-phase microextraction performances. Seven factors (or variables), i.e., volume of extraction, salt concentration, stirring speed, temperature, extraction time, type fiber sorbent and desorption time, were screened using a 2^7–3^ fractional factorial design. Fractional factorial design using a quite high number of variables is useful to reduce the number of individual experiments while limiting loss of significant information. For instance, while a full factorial design using seven factors requires at least 128 experiments, the 2^7–3^ fractional factorial design allows running “only” 32 experiments. The levels of each factor were chosen according to the results obtained during preliminary studies carried out by univariate optimization (data not shown). The levels of the factors and the related experimental domain are given in Table 1, the type of fiber being a qualitative variable, −1 represents C_18_ fiber, and +1 the Porapak Q one. The responses for each experiment are the surface areas measured for each peak assigned to analytes. Optimization aims at obtaining the larger surface areas.

**Figure 3:**
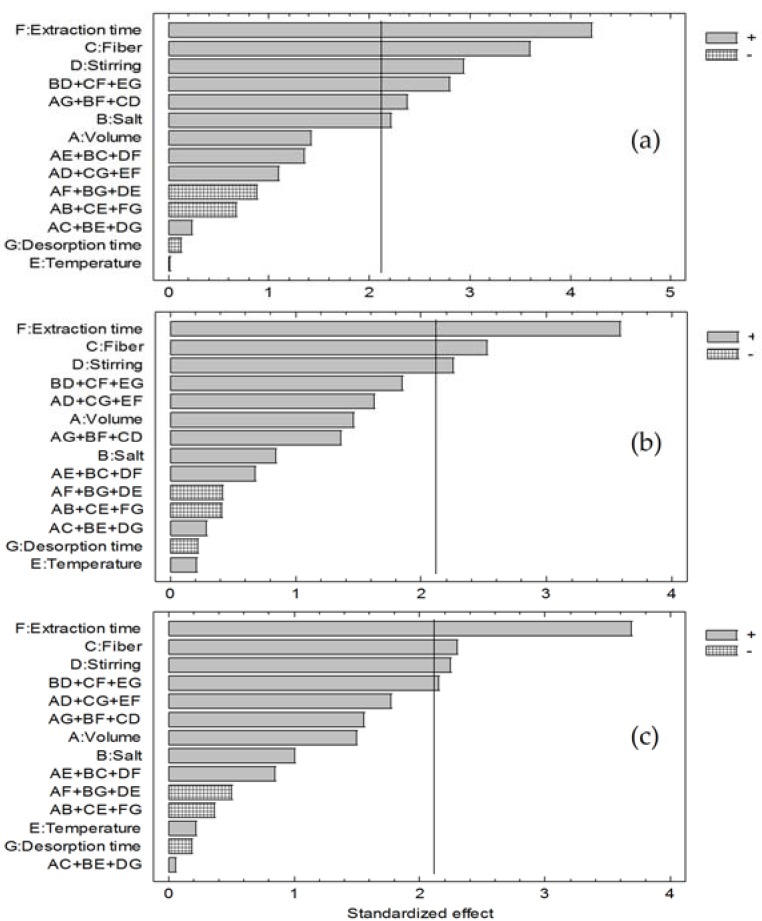
Pareto charts of the studied effects from the fractional factorial design 2^7–3^ for (**a**) clofibric acid; (**b**) ketoprofen; and (**c**) naproxen.

The mathematical correlation between analytical response and the variables was performed by analysis of variance (ANOVA). The Pareto chart that compare the effect of each factor (Figure 3) shows that the variables that were significant (*p*-value < 0.05) in the response of clofibric acid were extraction time, stirring speed, type of fiber and salt concentration, while, for ketoprofen and naproxen, significant variables were extraction time, stirring speed and type of fiber. 

A positive value for the estimated effect indicates an increase in the response if the variable increases to its high level. A negative value indicates that a better response is obtained at low levels of the variable. With respect to the influence of the type of fiber on the response, being a qualitative variable, Porapak Q fiber was selected for obtaining higher analytical response than C_18_-functionalzed particle fiber. No significant interaction term common to the three analytes are observed. Therefore, the variables to consider in the next step of optimization microextraction are the following three variables: stirring speed, extraction time and NaCl concentration.

The following screening experiment allowed reducing the number of significant variables to be investigated. A more detailed study through a response surface method, more specifically a Box-Behnken design, was then realized. Table 2 shows the assessed levels for the three selected variables. The results of this design were evaluated by ANOVA. The *p*-value obtained for all three analytes were greater than 0.05, and correlation percentage values were higher to 70%, indicating that the Box-Behnken model seems suitable to describe the experimental data at a confidence level of 95%.

**Figure 4:**
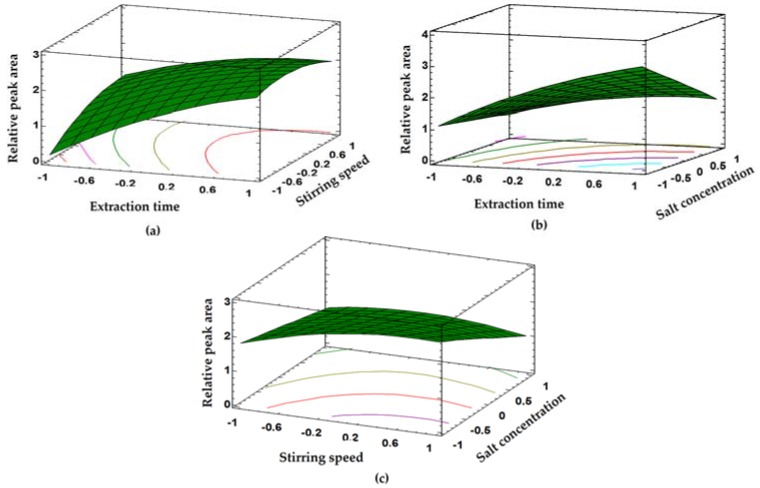
Response surfaces obtained from Box-Behnken design for naproxen: (**a**) extraction time vs. stirring speed; (**b**) extraction time vs. concentration of NaCl; and (**c**) stirring speed vs. concentration of NaCl.

Figure 4 shows the response surfaces obtained for the naproxen. From this response surfaces, it can be deduced that the analytical signal increases slightly with increasing stirring speed. Higher speeds were not evaluated because the vortex generated by the stirring does not allow a properly contact between the fiber and the sample. Therefore, a stirring speed of 600 rpm was selected.

NaCl was used to promote salting-out effect [31,35]. Under our experimental conditions increase in concentration of NaCl in the sample lead to reduction of the peak area of analyte. This effect is more pronounced for naproxen. It can be assume that increase of the salt concentration increases the viscosity of the sample solution, lowering the mass transfer of analytes from the sample solution onto the fiber. Therefore, NaCl will not be added to samples and standard solutions [25,26,36]. In addition, the response is function of extraction time. This is consistent with the already reported results on the extraction of other type of analytes. However, prolonged exposure times make the method tedious and lengthy. Thus, 45 min was selected as maximum extraction time that allows a compromise between good analytical signals and moderate times of analysis [25,26,36]. For ketoprofen and clofibric acid, the surfaces of responses were similar to those of naproxen (data not shown).

**Figure 5:**
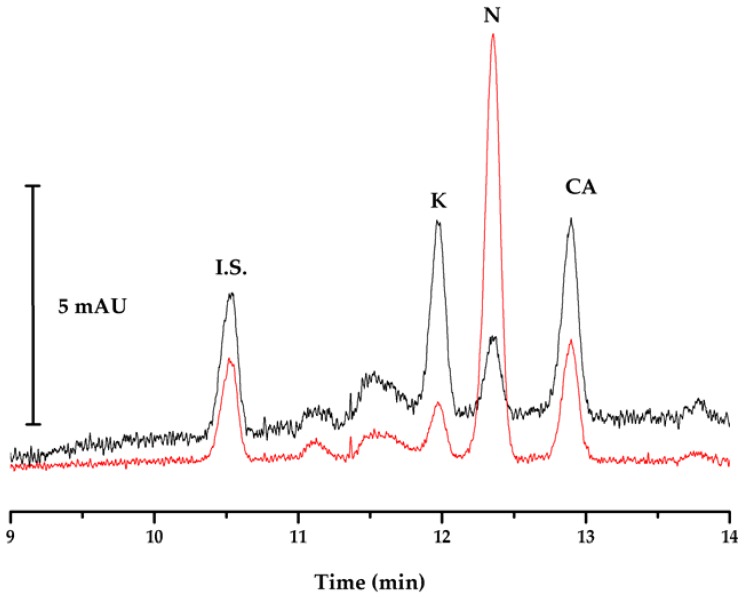
Typical electropherogram obtained under optimum conditions of a standard 30 µg∙L^−1^ solution of ketoprofen (K), naproxen (N), clofibric acid (CA), and internal standard (I.S.), at 203 (black) and 228 (red) nm.

Based on these results, the optimized experimental conditions for the developed method are summarized in Table 4. Figure 5 illustrates a typical electropherogram obtained from a mixture of 20 µg∙L^−1^ of each analyte using optimized SPME and electrophoretic separation conditions.

**Figure 6:**
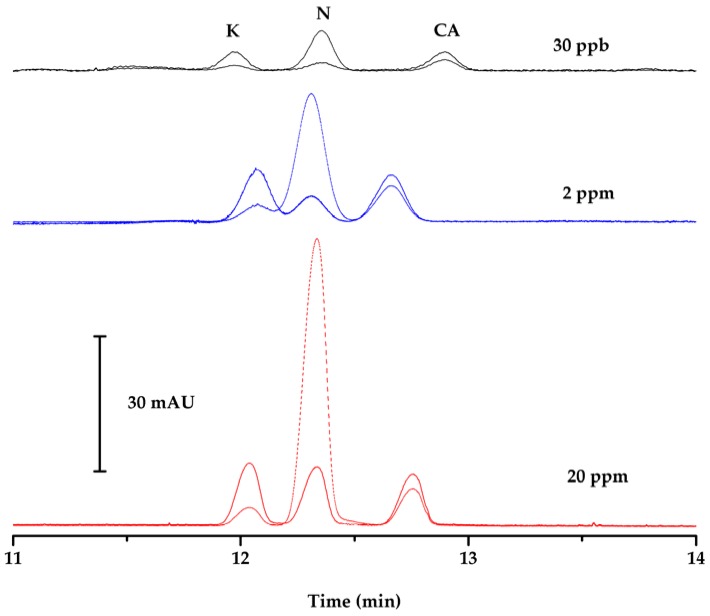
Electropherograms obtained under optimum conditions of standard solutions of ketoprofen (K), naproxen (N), clofibric acid (CA) at 20 mg∙L^−1^ without SPME (solid-phase microextraction) and ACN (acetonitrile) stacking (red), at 2 mg∙L^−1^ with ACN stacking (blue), and at 30 µg∙L^−1^ after SPME and ACN stacking (black), at 203 nm (straight line) and 228 nm (point segmented line).

It is worth noting that an approximately 25-fold increase in surface area of analyte peaks has been generated using the Porapak Q fiber for off-line preconcentration at optimized conditions of SPME. These results led to an estimation of combined preconcentration factor of 112 for naproxen, 234 for ketoprofen and 315 for clofibric acid when acetonitrile (ACN) stacking and off-line SPME with this laboratory-made fiber are coupled to capillary electrophoresis with diode array detector (CE-DAD), as shown in Figure 6.

### 3.4. Analytical Parameters

Calibration curves were obtained by preparing standard solutions at 4, 8, 20, 30 and 50 µg∙L^−1^ for ketoprofen and clofibric acid, and 3, 6, 20 30 and 50 µg∙L^−1^ for naproxen. Table 5 summarizes the main statistical parameters. R^2^ > 0.99 were obtained for the three calibration curves. The linear regression of each calibration curve was verified by *p*-value test.

The sensitivity for each analyte is determined by the slope of the calibration curves. The precision of the analytical method was estimated through intra-day reproducibility assay by performing five replicate analyses. Values lower than 13.3% were obtained. The limits of detection (LOD) and quantification (LOQ) of the presented method was calculated using Equations (1) and (2), respectively.

(1) $$\mathrm{LOD}=3\sigma /S_{a}$$

(2) $$\mathrm{LOQ}=10\sigma /S_{a}$$

where σ is the standard deviation of the analytical signals measured from 20 replicate at concentration of 4 µg∙L^−1^ for ketoprofen and clofibric acid; and 3 µg∙L^−1^ for naproxen, and S_a_ by the slope of the calibration curve.

### 3.5. Effect of the Matrix on the Extraction and Application of the Analytical Method to River Water Sample

In order to determine the presence or absence of matrix effect of real samples on the extraction method, two analytical curves using the optimized conditions were constructed for each analyte: one with the standard solution (calibration curve) and the other with water samples from the Genil River, Spain (standard addition). A statistical study (Student t) comparison was made between the slopes of the two curves. For ketoprofen and clofibric acid, t_calculated_ > t_critical_ and consequently there is matrix effect, therefore quantification of all analytes was performed by the standard addition method since they were simultaneously analyzed. For the validation of the developed analytical method, a recovery study of acidic drugs was performed on water samples from Genil River. Quantification was performed by standard addition and five measurements were realized for each level. The results of the recovery study for each analyte and the statistical parameters evaluated are given Table 6.

**Figure 7:**
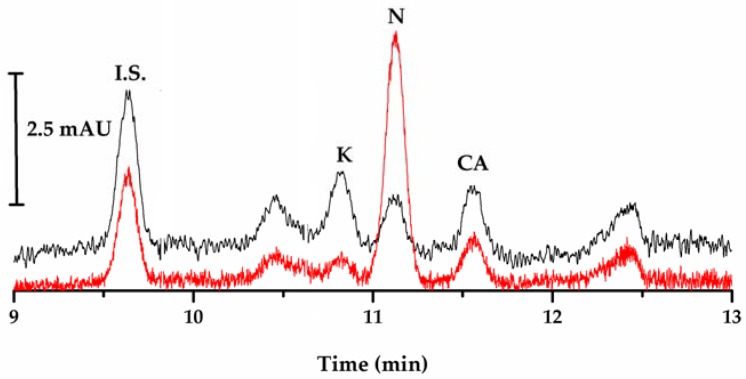
Typical electropherogram obtained under optimum conditions and after off-line SPME with the laboratory-made fiber of a spiked Genil River water sample at 10 µg·L^−1^ of ketoprofen (K), naproxen (N), clofibric acid (CA), and internal standard (I.S.), at 203 nm (black) and 228 nm (red).

The recovery yields obtained from the Genil River water sample at three different spiked concentrations, were in the range of: 99.7–103.6% for clofibric acid; 97.7–104.9% for ketoprofen; and 97.9 to 101.6% for naproxen. A one-sample *t*-test was performed to define whether the experimental means is significantly different from a reference value set to 100%. The statistical evaluation of the results for a probability level of 95% returned *p*-values > 0.05 in all cases, which shows no significant differences between the determined recovery yields of different levels, indicating that the method has adequate accuracy. A typical electropherogram obtained from a spiked Genil River water sample at 10 µg·L^−1^ of each analyte, after off-line SPME with the laboratory-made fiber is shown in Figure 7.

## 4. Discussion

The validation of this off-line solid-phase microextraction (SPME) coupled to capillary zone electrophoresis (CZE), acetonitrile (ACN) stacking and diode array detection (DAD) method (SPME-ACN Stacking-CZE-DAD) shows the possibility of accurate and precise analysis of ketoprofen, naproxen and clofibric acid in natural water bodies. It is well-known that capillary electrophoresis is a powerful separation technique but that its sensitivity (i.e., ultraviolet detection) can be seen as its Achilles heel; however, this off-line SPME and ACN stacking coupling improves reported limits of detection (LODs) in the literature when using capillary electrophoresis (CE) with diode array detection (CE-DAD) methods with other on-line preconcentration techniques [15,16,17,18,37], reaching at a combined preconcentration factors between 100 and 300 fold for the analytes of interest. In the literature, when LODs have reached levels under µg∙L^−1^ for these micropollutants in natural water samples, high performance liquid chromatography (HPLC) and gas chromatography (GC) methods coupled to high sensitivity detectors such as mass spectrometry (MS) and MS/MS, are developed [6,7,9,10,13,14,21,23,28,38,39,40], which indicates that using this type of detection is one perspective of this off-line SPME-ACN Stacking-CZE method in order to reach lower LODs.

Key feature of this method is that, to our point of view, off-line SPME coupled to CE-DAD in order to analyze acidic drugs in water samples has not been investigated yet. In fact, off-line solid-phase extraction is the technique mostly used in the developed methods with CE-DAD in detriment of using over 1 L of sample and high volumes of solvents, while with this off-line SPME allows to use only 5 mL of the sample and a less than a ten of milliliter of solvents per analysis. This advantage indicates that the off-line SPME with this laboratory-made fiber coupled to CE may be useful for other type of applications where the sample volume is a limitation, reducing the cost associated to the use of expensive reagents and limiting the exposition of manipulators to toxic ones [41,42]. In this regard, the analysis of other polar micropollutants in different aqueous samples may be another possible perspective to target.

Moreover, the precision of the method (Table 5) is in agreement with values reported in the literature when working with SPME [11,17,20,21,23,26,27]. Likewise, comparing to previously developed methods coupling off-line SPME to CZE analysis, the intra-day reproducibility (approximately 10%) of the new proposed method herein, is not significantly different [19,43,44]. It is worth noticing that such intra-day reproducibility values are also consistent with other coupled methods, i.e., SPME-HPLC or SPME-GC [20,21,23].

It is important to highlight, that the great advantage of using laboratory-made fibers, additional to the use of CE, is the low cost per analysis, as the price of these fibers is very low compared to the current value of a commercial SPME fiber, representing a considerable reduction in the cost of analysis. Moreover, the use of noncommercial SPME fibers is at least 100 times more economic than SPE, since only 5 mg of the sorbent is used to build a SPME fiber, while SPE cartridges requires the use of 500 mg of chromatographic support. Further advantages of these noncommercial fibers are high sensitivity, using only microliters of acetonitrile in desorption of analytes, using low sample volume and reuse of the same fiber up to more than 70 extractions.

One other advantage of using these fibers is the facile and rapid fabrication procedure of this laboratory-made Porapak Q fiber by physical deposition (more than 100 fibers has been prepared), when compared to those published in the literature, such as molecular imprinted polymerization [29,45,46], aluminum based metal-organic framework organic monolithic polymerization [30], ionic-liquid crosslinked polymerization [23], sol-gel technology [19,21], etc., which are time consuming and complicated in terms of the needed materials and the several steps to follow.

## 5. Conclusions

A new analytical method enabling the simultaneous determination of acidic drugs ketoprofen, naproxen and clofibric acid in aqueous media coupling off-line solid-phase microextraction with a new Porapak Q fiber, on-line preconcentration by acetonitrile stacking, and Capillary Zone Electrophoresis separation was developed. CE analysis allows baseline separation of the three acidic drugs within 12 min. 

The protocol of fiber synthesis is a low-cost and fast process. The extraction parameters were optimized using response surface methodology and was validated and applied to samples of the Genil River. This method represents an economic alternative for extraction and analysis of highly polar compounds from water samples. The method provides adequate accuracy and precision of analysis. 

## Figures and Tables

**Table 1:** Variables and levels used for fractional factorial design 2^7–3^.

Variable	Code	Level	
		Low (−1)	High (+1)
Sample volume (mL)	A	4	25
Salt concentration, NaCl (g∙mL^−1^)	B	0	0.15
Sorbent type	C	C_18_	Porapak Q
Stirring speed (rpm)	D	300	600
Extraction temperature (°C)	E	25	45
Extraction time (min)	F	15	30
Desorption time (min)	G	2	10

**Table 2:** Variables and levels used for Box-Behnken design.

Variable	Code	Level		
		−1	0	1
Extraction time (min)	A	15	27.5	40
Stirring speed (rpm)	B	300	450	600
Salt concentration, NaCl (g∙L^−1^)	C	0	0.075	0.15

**Table 3:** Experimental resolution values in function of the injection time.

t_inj_ (s)	R_S K-N_	R_S N-CA_
10	6.6	4.6
50	1.21	2.05
70	0.89	1.74
100	No separation	

t_inj_: injection time at 50 mbar; R_S K-N_: peaks resolution between ketoprofen and naproxen; R_S N-CA_: peaks resolution between naproxen and clofibric acid.

**Table 4:** Optimized variables of solid-phase microextraction.

Variables	Optimal Condition
Salt concentration, NaCl (% *m/v*)	0
Sample volume (mL)	5
Extraction time (min)	45
Stirring speed (rpm)	600
Extraction temperature (°C)	25
Type of fiber (sorbent)	Porapak Q
Desorption time (min)	4

**Table 5:** Analytical parameters of the studied acidic drugs using SPME-Acetonitrile Stacking-CZE-DAD.

Parameter	Clofibric Acid	Ketoprofen	Naproxen
*Calibration curve*			
Linear dynamic range (µg·L^−1^)	4–50	4–50	3–50
Intercept (a)	0.0758	0.0372	−0.0071
S_a_	1.49 × 10^−2^	5.78 × 10^−3^	2.08 × 10^−2^
Slope (b)	0.0321	0.0160	0.0548
S_b_	6.30 × 10^−4^	2.47 × 10^−4^	8.83 × 10^−4^
R^2^	0.9956	0.9974	0.9970
Linearity (regression coefficient, R)	99.12	99.48	99.41
*Intra-day reproducibility (%RSD), n = 5*			
6 µg∙L^−1^	-	-	12.8
8 µg∙L^−1^	13.3	8.9	-
20 µg∙L^−1^	10.3	9.9	9.8
50 µg∙L^−1^	8.5	9.8	9.5
*Limit of detection* (µg∙L^−1^)	0.96	1.27	0.98
*Limit of quantification* (µg∙L^−1^)	2.91	3.86	2.96

a: Intercept; S_a_: Standard deviation of intercept; b: Slope; S_b_: Standard deviation of slope;r: Coefficient de correlation. SPME: solid-phase microextraction; CZE-DAD: capillary zone electrophoresis with diode array detection.

**Table 6:** Recovery study results in Genil River water sample.

Analyte	Spiked Concentration (µg∙L^−1^)	Found Concentration (µg∙L^−1^)	Recovery (%)	$\bar{X}$(%) ± s ^a^	t	*p* ^b^
Clofibric acid	8	8.2	103.4	103 ± 3	2.605	0.051
		8.5	106.6			
		8.3	103.7			
		8.1	101.7			
		7.9	99.5			
	16	14.6	91.3	99 ± 5	−0.131	0.902
		16.4	102.9			
		16.5	103.4			
		16.0	100.4			
		16.0	100.4			
	20	20.4	102.4	103 ± 4	1.889	0.132
		21.2	106.4			
		19.3	96.8			
		20.9	104.7			
		21.5	107.5			
Ketoprofen	8	8.4	105.8	105 ± 5	2.079	0.106
		8.6	108.2			
		8.1	101.8			
		8.7	109.7			
		7.8	97.5			
	16	16.0	100.3	103 ± 4	1.707	0.163
		16.0	100.2			
		16.1	101.1			
		16.4	103.0			
		17.3	108.5			
	20	19.3	96.7	98 ± 6	−0.883	0.427
		18.9	94.5			
		19.6	98.0			
		18.5	92.5			
		21.4	107.1			
Naproxen	8	8.2	102.9	95 ± 17	−0.654	0.549
		9.0	112.8			
		8.0	100.1			
		7.3	91.4			
		5.4	67.5			
	16	15.3	95.8	99 ± 4	−0.359	0.737
		16.1	100.9			
		16.8	105.1			
		15.7	98.1			
		15.5	96.8			
	20	22.2	111.4	101 ± 8	0.446	0.679
		21.1	105.8			
		20.9	104.5			
		18.8	94.2			
		18.4	92.0			

^a^ Average value ± standard deviation, *n* = 5; ^b^ Found value in the test of comparison of an experimental mean with a reference value.

## Supplementary Materials

The following are available online at www.mdpi.com/1660-4601/14/7/739/s1, Figure S1: Electropherograms obtained after off-line SPME with laboratory-made fibers prepared with different sorbents, of a standard solution of ketoprofen (K), naproxen (N), clofibric acid (CA), and internal standard (I.S.), at 203 (blue) and 228 (red) nm; Table S1: SPME conditions with fibers prepared with different sorbents.
