# Supplementary material for: Development of a New Microextraction Fiber Combined to On-Line Sample Stacking Capillary Electrophoresis UV Detection for Acidic Drugs Determination in Real Water Samples
Source: Int J Environ Res Public Health. 2017 Jul 7;14(7):739. doi: 10.3390/ijerph14070739 (PMC5551177; doi:10.3390/ijerph14070739)
Supplement: Supplementary file 1 [file ijerph-14-00739-s001.pdf]

International Journal of
Environmental Research and Public Health

MDPI

Supplementary Materials: Development of a new microextraction fiber combined to online sample stacking capillary electrophoresis UV detection for acidic drugs determination in real water samples

The selection of the appropriate sorbent is an important step in SPME methodology, since it depends on the chemical nature of the studied drugs. For its selection, certain aspects should be considered, such as: chemical affinity between sorbent and analytes, adequate volume of the stationary phase, and mechanical, thermal and chemical resistance of the fiber [1–3].

The sorbents that were tested were: C_{18}, graphitized carbon, LiChrolut, Chromosorb P and Porapak (Q, QS, R and N). The fiber with C_{18} was tested at two thicknesses, one of 0.9 millimeters (thin) and one of 1.5 millimeters (thick). For SPME, samples of deionized water at pH 2 containing ketoprofen, naproxen and clofibric acid at 50, 37.5 and 75 $\mu g.L^{-1}$ respectively, were used. Each extraction was performed in triplicate under the conditions described in Table S1.

Table S1. SPME conditions with fibers prepared with different sorbents.

Variables	Value
Salt concentration, NaCl (% m/v)	0.01
Sample volume (mL)	25
Extraction time (min.)	20
Stirring speed (rpm)	600
Extraction temperature (ºC)	25
Desorption time (min)	10
Volume of desorption solution (µL)	100

The graphitized carbon fiber lost part of the coating during desorption of the analyte as a result of the large particle size, which conferred it a low mechanical resistance. On the other hand, capillary clogging was found when using the thick fiber of C_{18}, which could have been due to particles of the stationary phase being released to the desorption solution when shocks occur between the fiber and the vial insert during sonication as a product of its thickness. Therefore, both fibers were discarded as part of the group of fiber sorbents to be investigated. The electropherogram corresponding to each of the remaining stationary phase materials are shown in Figure S1. These electropherograms were obtained with a silica capillary of 75 µm I.D with an extended optical path, and picric acid was used as an internal standard.

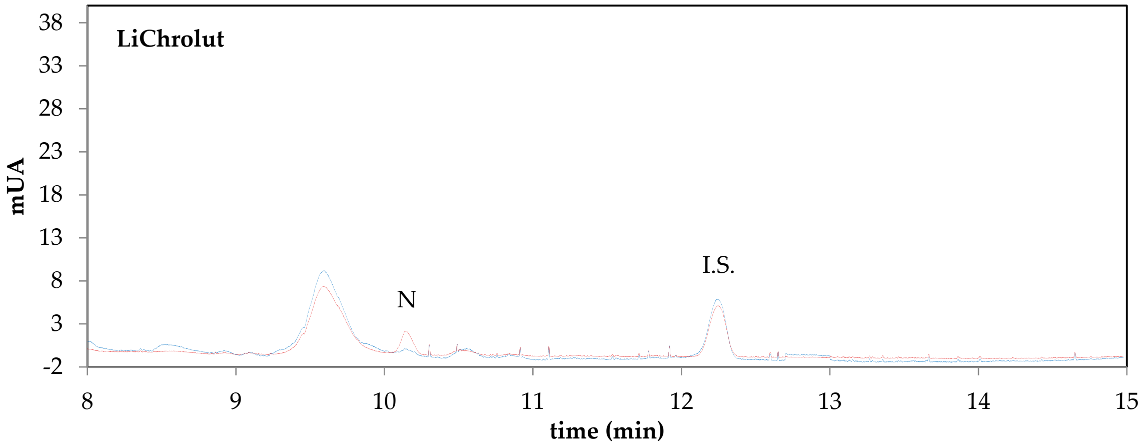
LiChrolut
mUA
38
33
28
23
18
13
8
3
-2
N
I.S.
8
9
10
11
12
13
14
15
time (min)

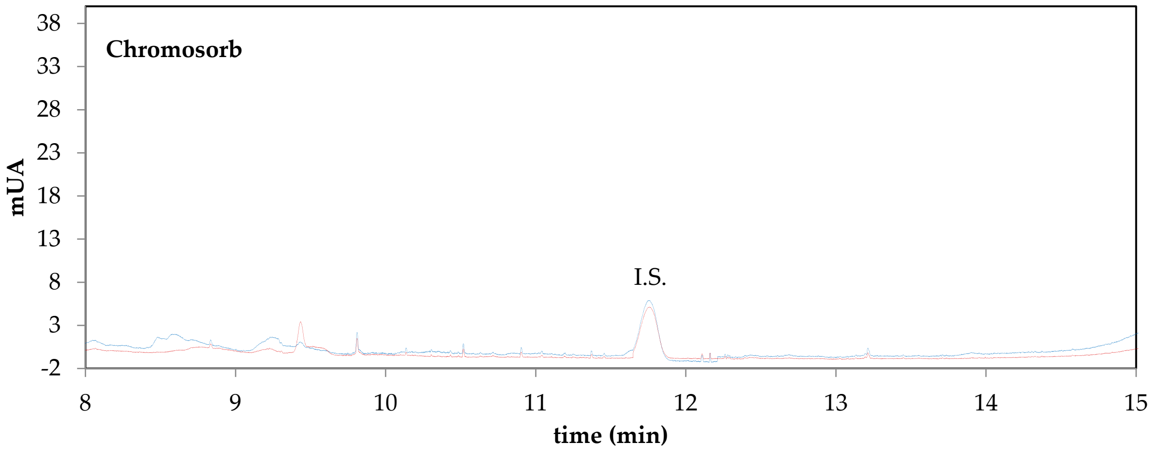
Chromosorb
mUA
38
33
28
23
18
13
8
3
-2
I.S.
8
9
10
11
12
13
14
15
time (min)

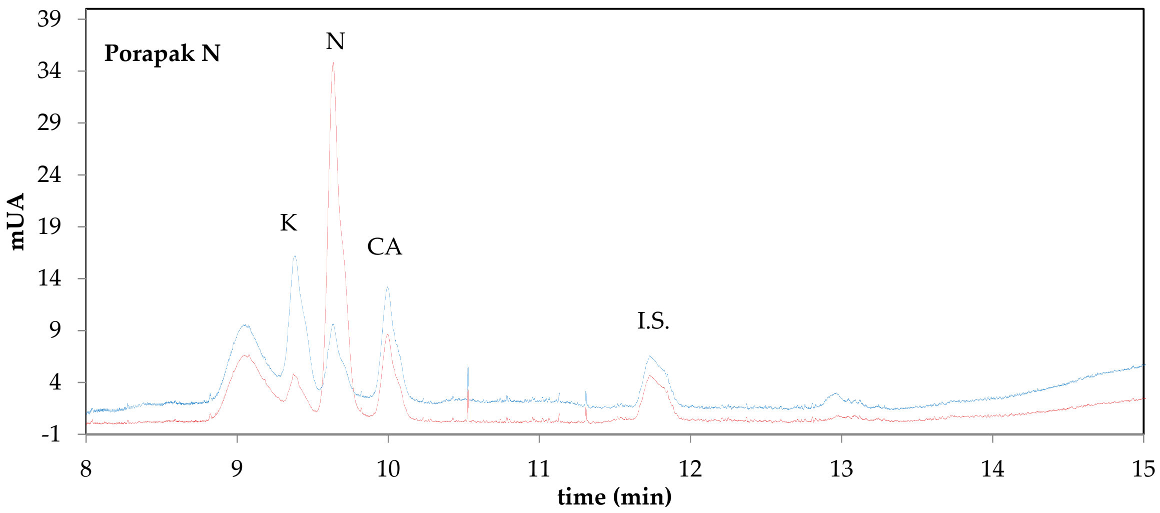
Porapak N
mUA
39
34
29
24
19
14
9
4
-1
K
N
CA
I.S.
8
9
10
11
12
13
14
15
time (min)

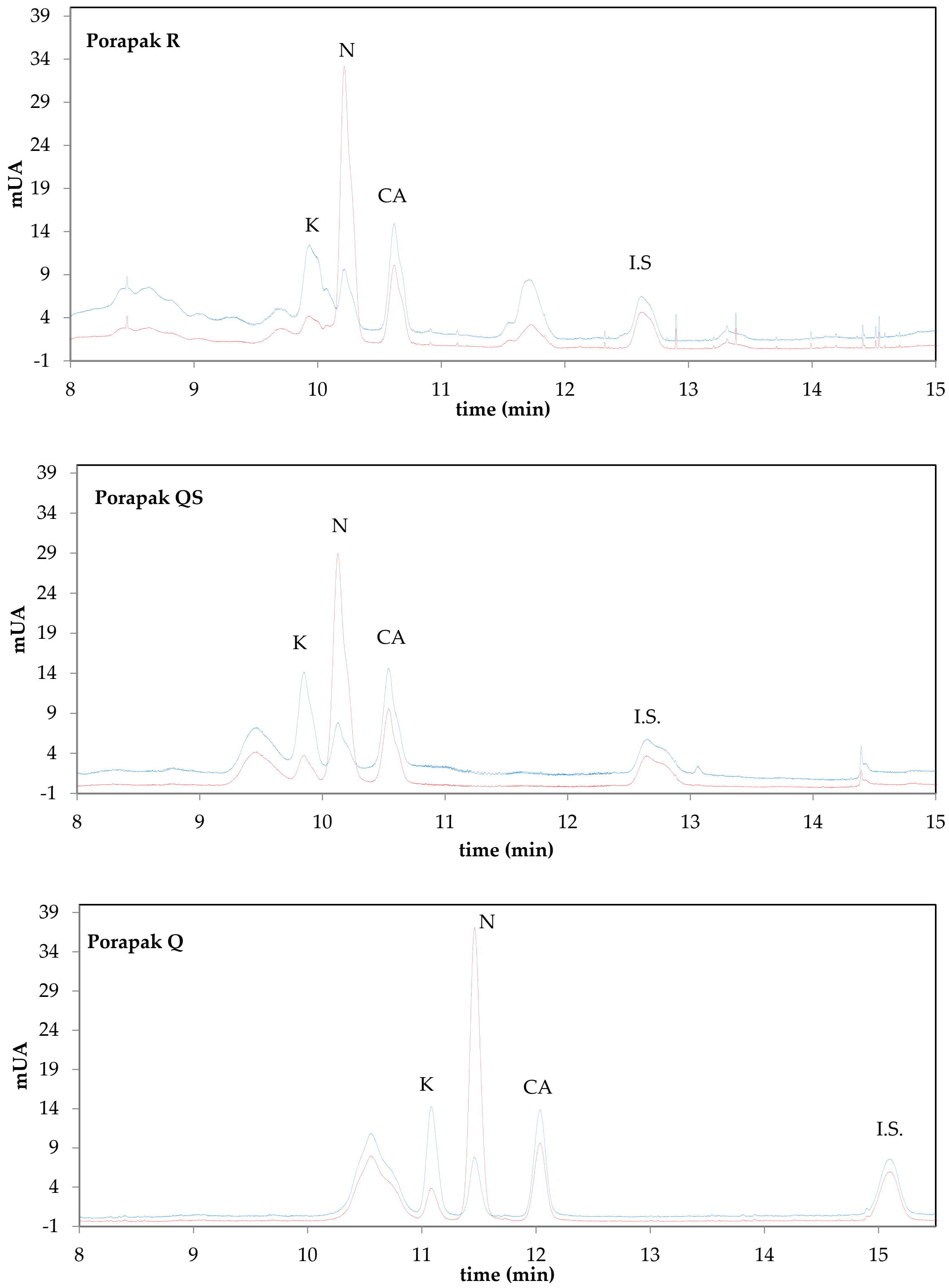
Porapak R
N
K
CA
I.S
mUA
time (min)
Porapak QS
N
K
CA
I.S.
mUA
time (min)
Porapak Q
N
K
CA
I.S.
mUA
time (min)

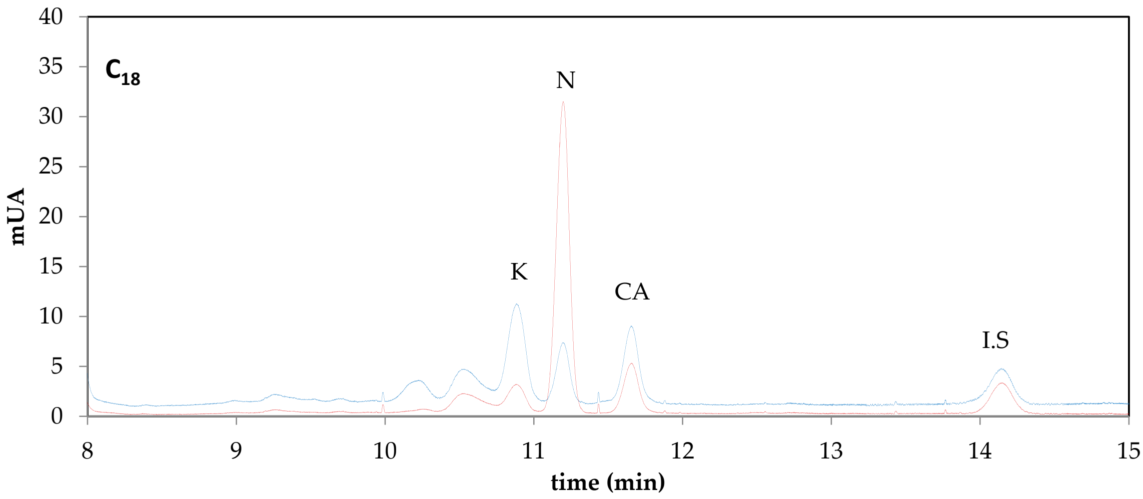

Figure S1. Electropherograms obtained after off-line SPME with laboratory-made fibers prepared with different sorbents, of a standard solution of ketoprofen (K), naproxen (N), clofibric acid (AC), and internal standard (I.S.), at 203 (blue) and 228 (red) nm.

As shown in Figure S1, with the materials LiChrolut and Chromosorb there was no extraction of study acidic drugs, whereas with the C_{18} and Porapak fibers the opposite is observed, which may be due to the affinity of these materials to analytes, which are in non-dissociated form due to the low pH of the sample [1–3]. On the other hand, among the sorbents of the Porapak series, the one that allowed a good extraction with the smallest quantity of interfering peaks and with more symmetrical peaks was the Porapak Q. Thus, the thin-C_{18} and Porapak Q fibers were selected during the multivariate optimization of SPME.

The great advantage of using fibers manufactured in the laboratory is the low cost of analysis, since considering that only 5 mg of the sorbent is used to build a SPME fiber, and that SPE requires the use of cartridges of 500 mg, then the SPME is at least 100 times more economical than the SPE[4].

References

1. Theodoridis, G.; Koster, E. H. M.; de Jong, G. J. Solid-phase microextraction for the analysis of biological samples. *J. Chromatogr. B. Biomed. Sci. App.* **2000**, *745*, 49–82, DOI:10.1016/S0378-4347(00)00203-6.
2. Ulrich, S. Solid-phase microextraction in biomedical analysis. *J. Chromatogr. A* **2000**, *902*, 167–194, DOI:10.1016/S0021-9673(00)00934-1.
3. Cela, R.; Rosa, A. L.; Casais, M. *Tecnicas de separacion en quimica analitica*; Sintesis: Madrid, Espana, 2002.
4. Macià, A.; Borrull, F.; Aguilar, C.; Calull, M. Improving sensitivity by large-volume sample stacking using the electroosmotic flow pump to analyze some nonsteroidal anti-inflammatory drugs by capillary electrophoresis in water samples. *Electrophoresis* **2003**, *24*, 2779–2787, DOI:10.1002/elps.200305542.

© 2017 by the authors. Submitted for possible open access publication under the terms and conditions of the Creative Commons Attribution (CC BY) license (http://creativecommons.org/licenses/by/4.0/).
